# Comparison of surgical and conservative treatment of Rockwood type-III acromioclavicular dislocation: A meta-analysis

**DOI:** 10.1097/MD.0000000000009690

**Published:** 2018-01-26

**Authors:** Guolong Tang, Yu Zhang, Yuan Liu, Xiaodong Qin, Jun Hu, Xiang Li

**Affiliations:** a Department of Orthopedics; b Department of Infectious Diseases, the First Affiliated Hospital of Nanjing Medical University, Nanjing, China.

**Keywords:** acromioclavicular dislocation, complication, conservative treatment, function, Rockwood type, surgical treatment

## Abstract

**Background:**

There is no consensus on the effects of surgical versus conservative treatment on Rockwood type-III dislocation of the acromioclavicular joint in general orthopedic practice. The objective of this meta-analysis was to compare the clinical outcomes of patients managed surgically and conservatively following type-III acromioclavicular (AC) dislocation.

**Methods:**

The Cochrane Library, EMBASE, MEDLINE via Ovid SP, and PubMed databases were searched for randomized controlled trials and observational studies. Patient data were pooled using standard meta-analytic approaches. For continuous variables, the weighted mean difference was used. For dichotomous data, the odds ratio was calculated.

**Results:**

The current analysis included 10 trials on this topic, and the results demonstrated that there were no significant differences between surgical and conservative treatment in terms of pain, weakness, tenderness, post-traumatic arthritis, restriction of strength, unsatisfactory function, and scores (Constant, UCLA, Imatani, SST, DASH, Larsen). Analyses of ossification of the coracoclavicular ligament (OR = 1.62, 95% CI = 1.01–2.61) and osteolysis of the lateral clavicle (OR = 2.87, 95% CI = 1.27–6.52) suggested better function with conservative treatment versus surgical treatment, but the latter was superior to conservative treatment with regard to loss of anatomic reduction. Only 1 study showed a higher acromioclavicular joint instability score for surgical management compared with conservative management (*P* < .00001).

**Conclusion:**

In the management of Rockwood type-III AC dislocation, conservative treatment is superior to surgical treatment. Nonoperative treatment results in a lower incidence of ossification of the coracoclavicular ligament and osteolysis of the lateral clavicle compared with operative treatment. However, there was no statistical difference between operative and nonoperative treatment in terms of clinical outcomes.

## Abstract 2

Supplemental Digital Content is available in the text

## 1 Introduction

Acromioclavicular (AC) dislocation is a common shoulder injury, and often occurs in contact sports. One study showed 40% of 226 collegiate football players with shoulder injuries had AC dislocation.^[1]^ A study in 233 quarterback players in the national football league of USA who had shoulder injuries also found the percentage of AC dislocation to be 40%.^[2]^

Rockwood proposed a classification system based on AC dislocation severity to allow patients to receive individualized therapy. In this classification system, “type III” is defined as AC dislocation accompanied by complete rupture of the acromioclavicular and coracoclavicular ligaments. In general, conservative treatment is used in Rockwood type-I and -II acute AC dislocation and surgical treatment is used for type-IV, -V and -VI AC dislocation.^[3]^ However, there is controversy over which treatment—conservative care or surgical intervention—is the better approach for type-III AC injuries. Studies have reported reasonable results after conservative care of acute injuries to the AC joint. Two prospective randomized controlled trials (RCTs) compared the clinical efficacy of surgical and conservative approaches in the treatment of acute AC dislocation. Larsen et al^[4]^ found that conservative treatment resulted in a relatively short recovery time; there was no significant difference in clinical outcomes between surgical and conservative groups at 13-month follow-up. Similarly, Bannister et al^[5]^ found that patients who received conservative treatment recovered faster and more comprehensively, and returned to work earlier. Moreover, there were fewer unsatisfactory outcomes. However, the authors of both studies agreed that patients with severe AC dislocation or those doing heavy manual labor may benefit from surgical treatment. Some scholars who support surgical treatment also believe that joint anatomic reconstruction is more reliable and achieves satisfactory clinical outcomes. Gstettner et al^[6]^ found the mean Constant score to be 80.7 in the conservatively treated group and 90.4 in the group that underwent surgery, and the mean coracoclavicular distance to be 15.9 mm and 12.1 mm, respectively.

In 2011, Smith et al^[7]^ selected 6 retrospective studies to compare the clinical outcomes of patients cared for by surgical and conservative means after type-III AC dislocation. They found that surgical treatment could have better clinical efficacy, but the recovery time was longer than that for conservative treatment. In addition, there were no significant differences in strength, pain, throwing ability, and the incidence of osteoarthritis of the acromioclavicular joint between patients receiving surgical treatment and patients receiving conservative treatment. Tamaoki et al^[8]^ selected 3 studies to carry out a meta-analysis. They found insufficient evidence from RCTs to ascertain if surgical treatment is indicated for AC dislocation in adults.

However, since that time, 2 randomized controlled trials^[9,10]^ and 1 retrospective trial^[11]^ comparing conservative care or surgical intervention in terms of clinical complications, outcomes, and functional shoulder scores have been published. Therefore, we believe it is necessary to update the information on this topic.

## 2 Methods

### 2.1 Search strategy and study selection

This meta-analysis was carried out following the guidelines of the PRISMA statement.^[12]^ We searched the Cochrane Library, EMBASE, and MEDLINE via Ovid SP and PubMed on April 10, 2016 with no limitations on date/time, language, document type, or publication status. Keywords were collected through expert opinion, literature review, controlled vocabulary (Medical Subject Headings = MeSH and Excerpta Medica Tree = EMTREE), and by reviewing the primary search results. Search strategies developed with assistance from a medical-information specialist were reported in Supplement 1 (data are not shown). Search results were de-duplicated in EndNote X5 and sent to 2 researchers (GT and YL) for screening. Two of the authors (GT and YL) independently reviewed the articles on separate occasions. In addition, a manual search of the references in all primary articles and relevant previously published reviews and meta-analyses in the English scientific literature was undertaken to ensure that no studies were missed. Inclusion criteria were: (a) studies had to compare surgical treatment with conservative treatment after an acute, closed type-III AC dislocation; (b) RCTs and nonrandomized controlled trials (nRCTs) were included. Exclusion criteria were: (a) surgical and conservative treatment were not shown in the same article; (b) data were duplicated; (c) usable data were not reported. The approval by an ethics institutional review board is not required for this study because human subjects were not studied.

### 2.2 Data extraction and statistical analyses

Each included study was carefully reviewed to extract as much data as possible. Clinical function including unsatisfactory function (only “poor” or “fair” categories), scores (Constant, University of California Los Angeles scale [UCLA], Imatani, simple shoulder test (SST), disabilities of the arm, shoulder and hand [DASH], Larsen, acromioclavicular joint instability [ACJI]) and complications (pain, weakness, tenderness, loss of anatomic reduction, post-traumatic arthritis, ossification of coracoclavicular ligaments, osteolysis of the lateral clavicle, and restriction of strength) were within the scope of this meta-analysis.

We used RevMan v5.3 (recommended by the Cochrane Database of Systematic Reviews) to undertake this meta-analysis. With RevMan, the strength of the association between surgical treatment and conservative treatment was measured by odds ratios (ORs) and 95% confidence intervals (CIs). Publication bias was reviewed visually with funnel plots. Statistical heterogeneity was evaluated using chi-squared tests and *I*^2^ statistics. For outcomes in which *I*^2^ < 50% or *P* < .05, a fixed-effects model was adopted. If these assumptions were not met, a random-effects model was adopted. For clinical scores and function (which are continuous), we employed a fixed-effects model and the inverse variance method. For complications (which are dichotomous), we employed a fixed-effects model and the Mantel–Haenszel method. In each case, a *P* < .05 was considered significant, and 95% CIs were calculated.

### 2.3 Risk of bias assessment

The methodological quality of each study was assessed using the risk-of-bias assessment tool outlined in the Cochrane Handbook for Systematic Reviews of Interventions (version 5.3).

## 3 Results

### 3.1 Search results and characteristics of selected studies

**Figure 1:**
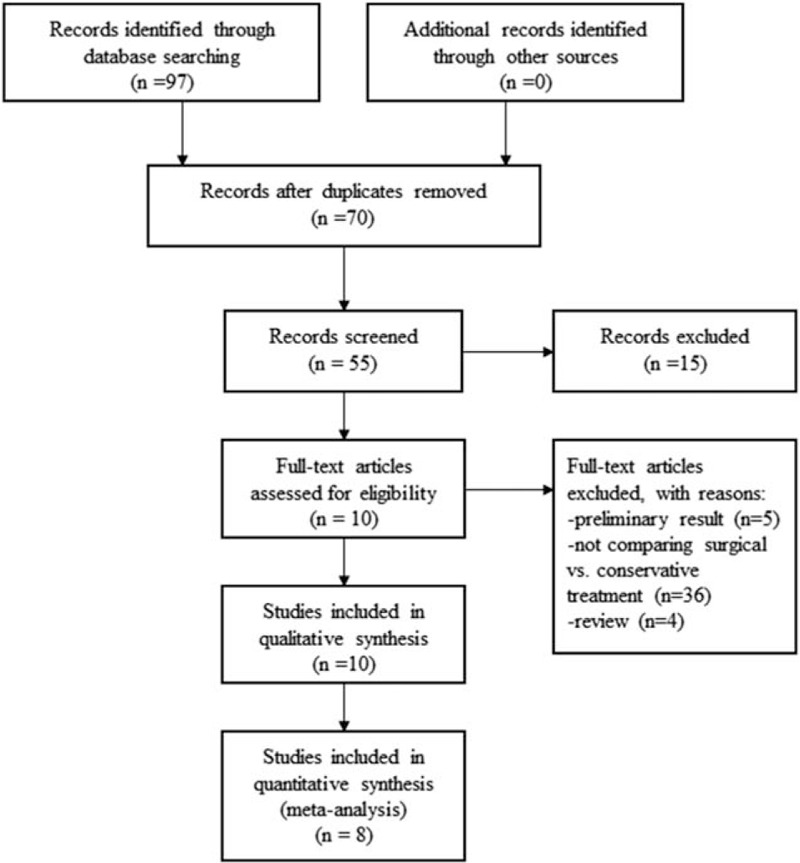
Flowchart of study selection and exclusion criteria from the present meta-analysis.

Details of search strategies are provided in Supplementary File 1. Ninety-seven articles were retrieved from 4 databases. The flowchart of study selection is shown in Figure 1. We included 10 studies^[4,6,9–11,13–17]^ involving 326 patients who underwent surgical treatment and 323 patients who underwent conservative management. The detailed characteristics of these studies are summarized in Table 1.

**Table 1:**
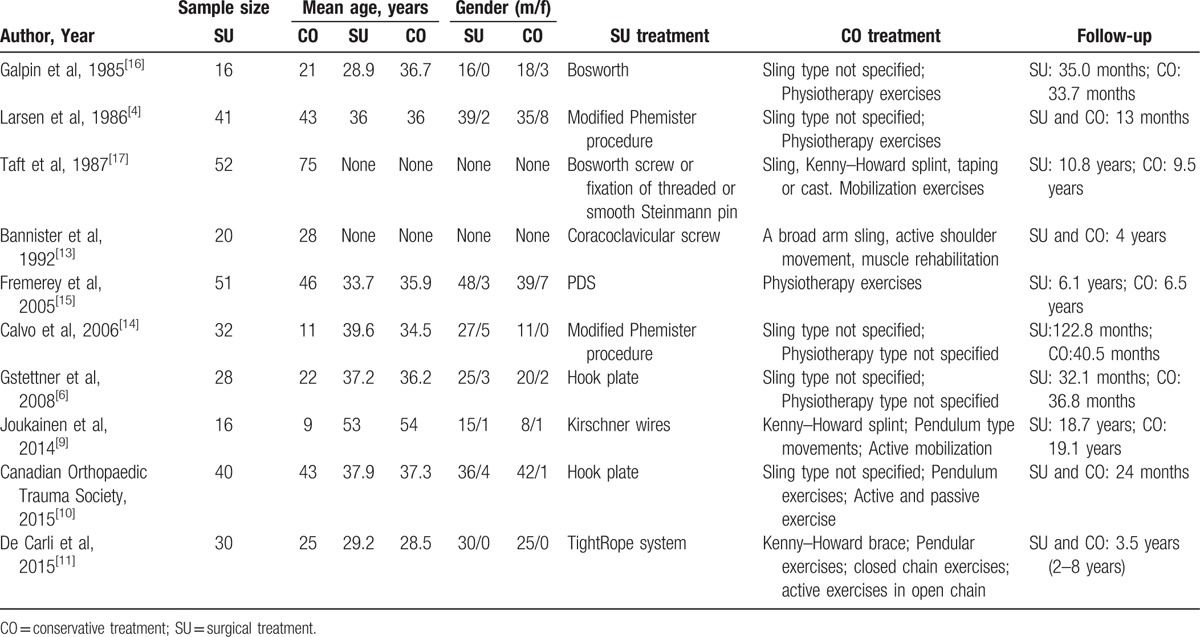
Basic characteristics of included studies.

### 3.2 Quality assessment and detection of publication bias

**Figure 2:**
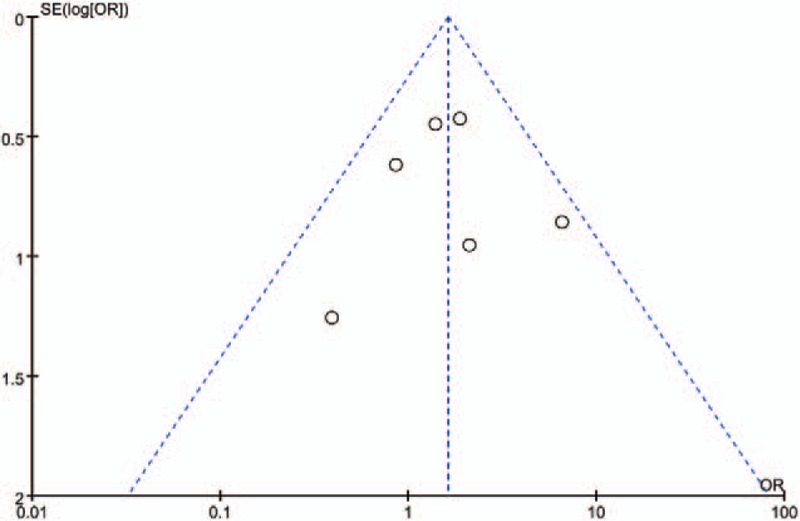
Funnel plot illustrating publication bias using the ossification of coracoclavicular ligament measure.

**Figure 3:**
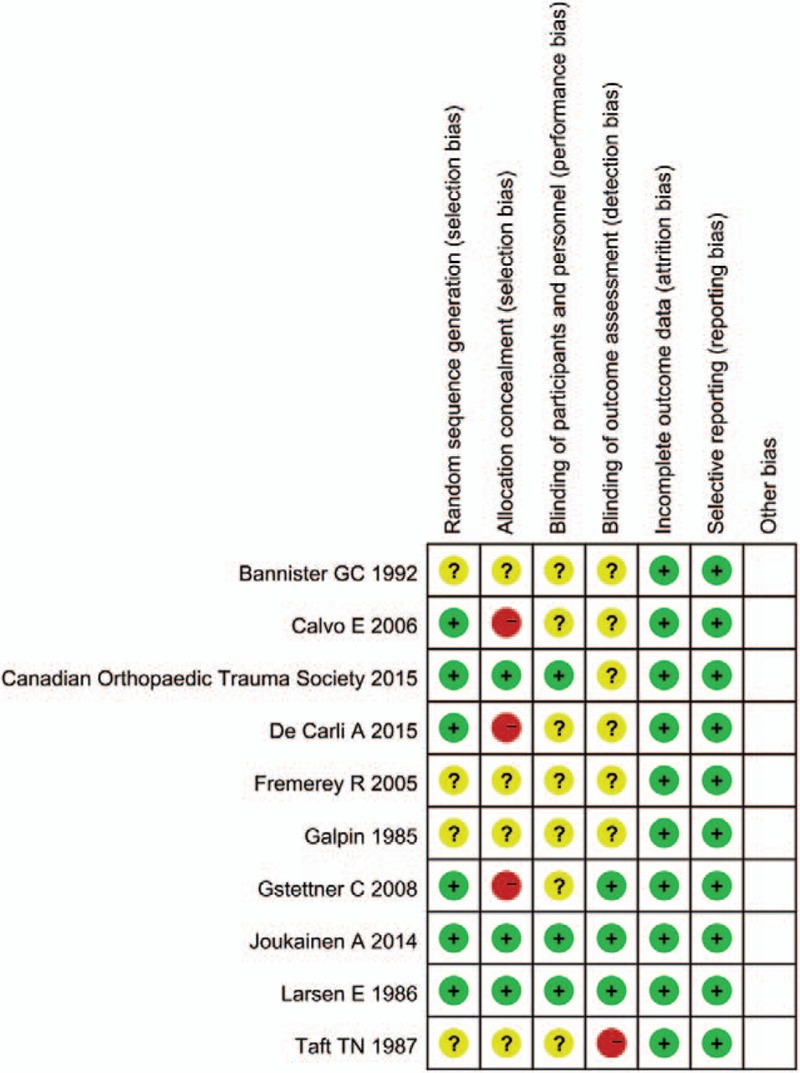
Quality assessment summary. Red color: high risk; yellow color: unclear risk; green color: low risk.

Quality assessment was carried out using a risk and bias table in RevMan and is summarized in Figure 2. Most of the articles were low-to-moderate risk with regard to quality assessment. Funnel plots were used to visually assess publication bias in RevMan (Fig. 3). We used analyses of ossification of coracoclavicular ligaments to generate this funnel plot because it included 6 of 10 studies and covered more than any other analyses. The data showed that there was no significant publication bias among these articles (Fig. 3).

### 3.3 Meta-analysis of complications

**Figure 4:**
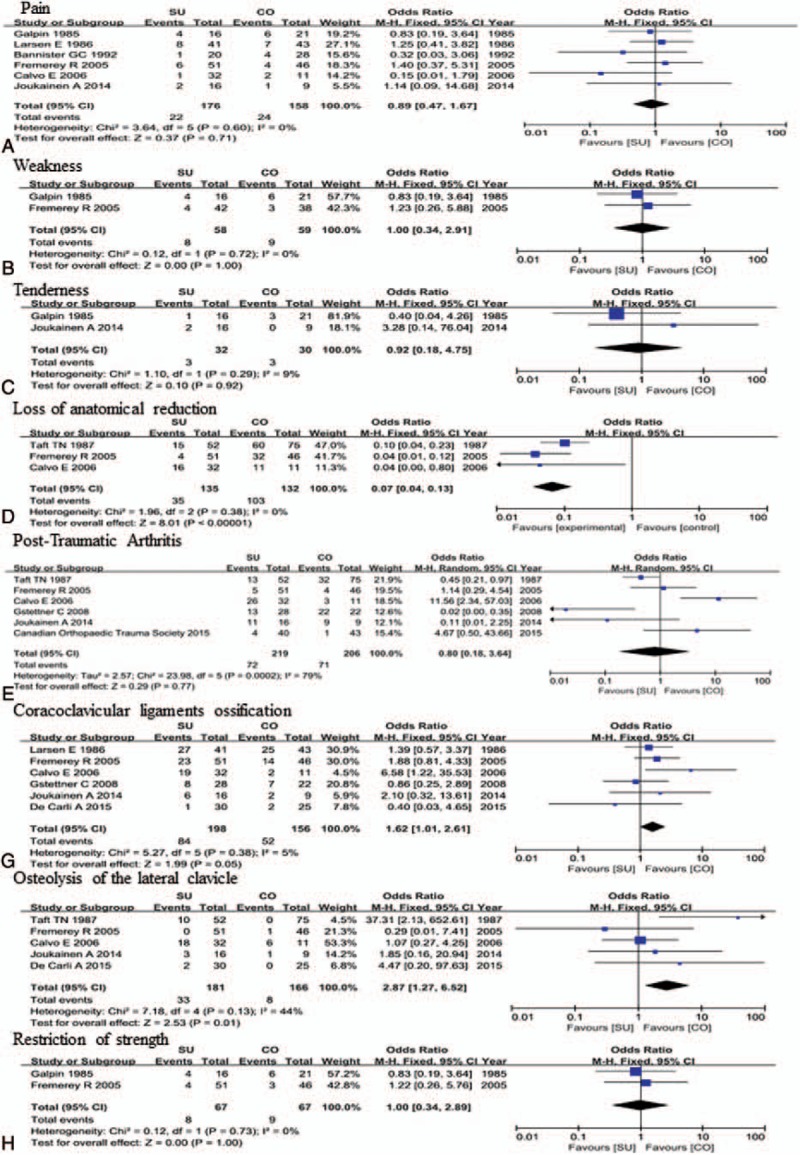
Meta-analysis of complications.

Meta-analysis of complications (pain, weakness, tenderness, loss of anatomic reduction, post-traumatic arthritis, ossification of coracoclavicular ligaments, osteolysis of the lateral clavicle, and restriction of strength) is shown in Figure 4 and Table 2. There was significant heterogeneity for post-traumatic arthritis (χ^2^ = 23.98, *I*^2^ = 79%, *P* = .0002), for which a random-effects model was employed. There were no significant differences among individual ORs for pain, weakness, tenderness, post-traumatic arthritis or restriction of strength. Ossification of coracoclavicular ligaments (OR = 1.62, 95% CI = 1.01–2.61) and osteolysis of the lateral clavicle (OR = 2.87, 95% CI = 1.27–6.52) analyses suggested better function with conservative treatment compared with surgical treatment. Three studies compared the change in loss of anatomic reduction and showed a lower prevalence of loss of anatomic reduction with surgical treatment (OR = 0.07, 95% CI = 0.04–0.13).

**Table 2:**
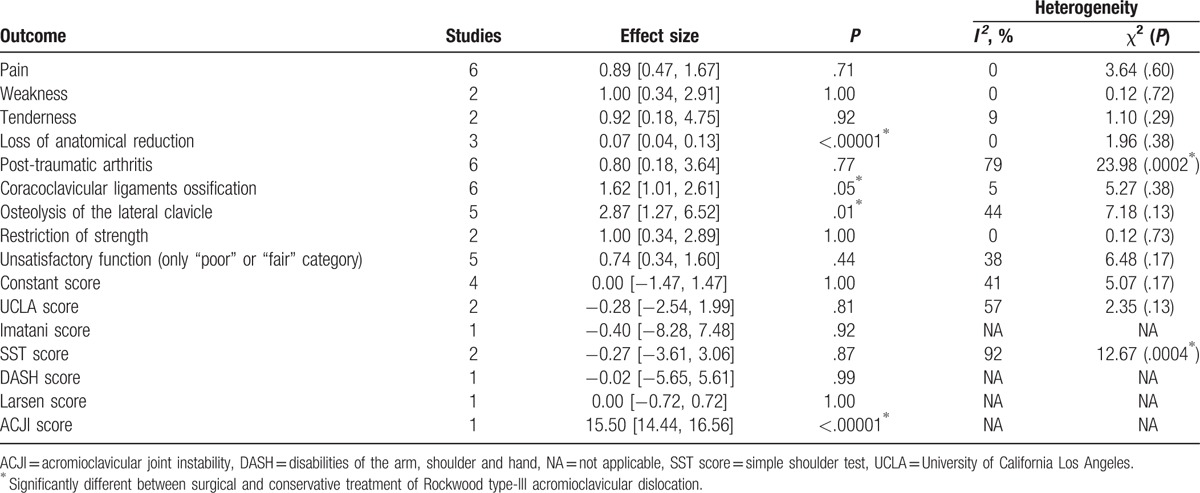
Results of the meta-analysis.

### 3.4 Meta-analysis of clinical function and clinical scores

**Figure 5:**
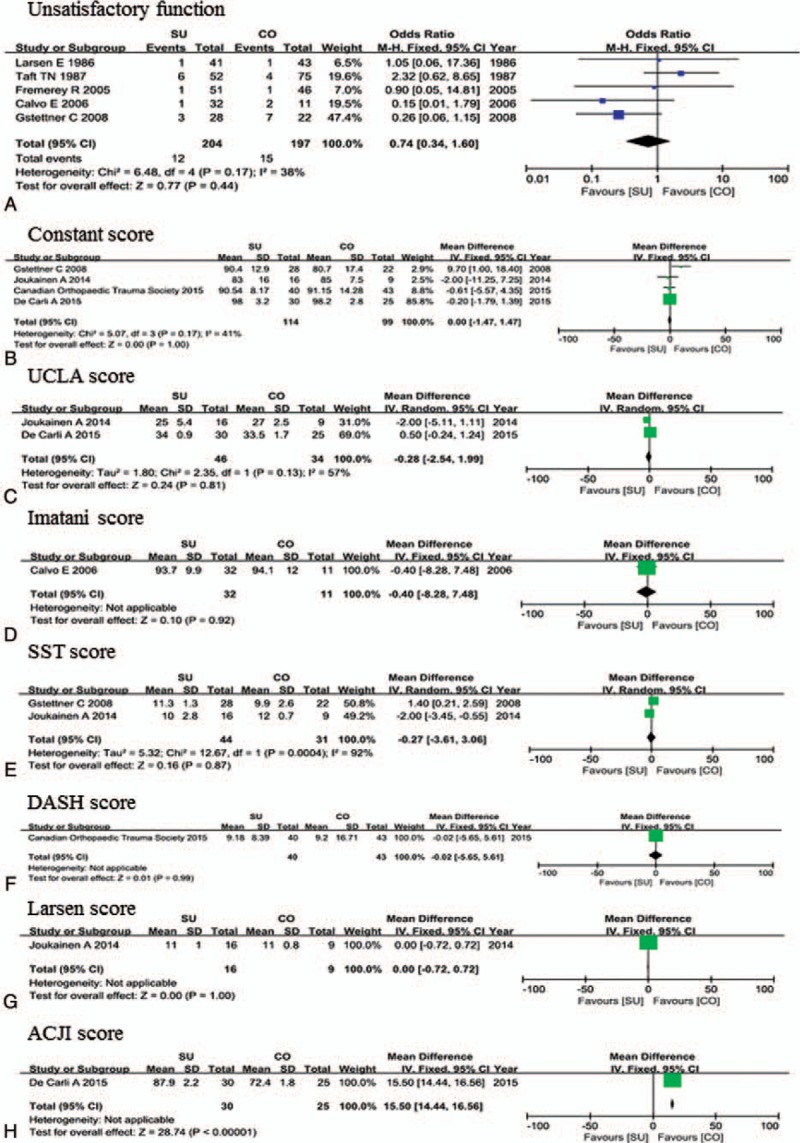
Meta-analysis of clinical function and clinical scores.

Meta-analysis of clinical function, including unsatisfactory function (poor or fair) and clinical scores (Constant, UCLA, Imatani, SST, DASH, Larsen, ACJI) are shown in Figure 5 and Table 2. Only 1 study analyzed Imatani, DASH, Larsen, and ACJI scores, so their heterogeneity was not applicable. Only meta-analysis of the SST score showed significant heterogeneity (χ^2^ = 12.67, *I*^2^ = 92%, *P* = .0004) and a random-effects model was employed. There were no significant differences between surgical treatment and conservative treatment among unsatisfactory function or Constant, UCLA, Imatani, SST, DASH, or Larsen scores. However, results for the ACJI score favored surgical treatment (OR = 15.50, 95% CI = 14.44–16.56) even though only 1 study included the ACJI score.

## 4 Discussion

In the present meta-analysis, conservative methods of treatment were slings, Kenny–Howard splint, taping, casts, and physiotherapy exercises. Surgical methods were the Bosworth clavicle–coracoid screw, Phemister procedure, coracoclavicular banding using resorbable polydioxanon thread, the Hook plate, and TightRope system. At present, treatment of type-III AC dislocation is controversial. Previously, conservative treatment was the main approach. Even though complete anatomic reduction by conservative treatment was difficult, functional recovery was good. Surgical treatment can be used to obtain complete anatomic reduction and minimize the risk of shoulder deformity.^[18,19]^ To compare the difference between these 2 approaches, by searching in a systematic manner, we selected 10 studies involved 649 patients with AC dislocation. Three out of these 10 studies were RCTs, 1 was a prospective study, and the other 6 were retrospective studies. We investigated the safety and efficacy of conservative and surgical approaches in the treatment of type-III AC dislocation by analyzing the prevalence of postoperative complications and function scores.

We found no significant difference in the prevalence of post-treatment osteoarthritis between surgical and conservative treatments. This meta-analysis comprised 6 studies involving 143 cases, of which 72 were in the surgical treatment group and 71 were in conservative treatment group. Taft et al^[17]^ found that 13 of 52 patients treated by surgery developed osteoarthritis; the prevalence of osteoarthritis in the surgical treatment group was lower than that in the conservative treatment group. The 2 surgical approaches, acromioclavicular pins and clavicle–coracoid screw, were used in that study. It seems that maintenance of the anatomic reduction of the acromioclavicular joint could reduce the risk of postoperative osteoarthritis. Studies by Gstettner et al^[6]^ and Joukainen et al^[9]^ support this view. The surgical approach adopted by Gstettner et al was a Hook plate and acromioclavicular Kirschner wires was adopted by Joukainen et al. However, the study by Calvo et al^[14]^ showed contrary evidence that the prevalence of osteoarthritis in the surgical treatment group was significantly higher than that in the conservative treatment group. The surgical approach adopted was a modified version of the Phemister procedure. It was hypothesized that the higher prevalence of osteoarthritis may have been associated with fixation of the acromioclavicular joint after reduction with a Kirschner wire. The other 2 studies suggested that the prevalence of post-traumatic osteoarthritis did not differ between the 2 groups. The adopted surgical approaches were coracoclavicular banding using resorbable polydioxanon thread and the Hook plate. The heterogeneity in this meta-analysis may have been related to differences in the surgical approaches. The heterogeneity is expected to decrease if the same surgical approach is compared.

The prevalence of ossification of the coracoclavicular ligament or osteolysis of the lateral clavicle was higher for surgical treatment than for conservative treatment. Ossification of the coracoclavicular ligament refers to the calcification spots and even cord-like calcification stripes in ligaments in radiographs image that are often observed in the later stages of ligament repair after AC dislocation. A recent research arose the concern over the occupying effect of the calcified ligament which might lead to subacromial impingement syndrome. The ossification of the coracoclavicular ligament might be 1 potential cause of shoulder pain and motion restriction.^[20]^ Osteolysis of the lateral clavicle refers to the osteolysis of the distal clavicle that may occur after acute shoulder injury or which is caused by repeated micro-trauma to the shoulder. It is characterized by gradual reabsorption of the lateral clavicle. The most common symptoms are pain and swelling at the acromioclavicular joint. Osteolysis of the lateral clavicle may be related to disorders in the microenvironment of the distal clavicle, but the specific mechanism is not known.^[21]^

For evaluation of the recovery of shoulder function after surgical or conservative treatments, we found no significant differences between surgical treatment and conservative treatment among the domains of unsatisfactory function as well as Constant, UCLA, Imatani, SST, DASH, and Larsen scores. However, analyses of the ACJI score favored the surgical group, though only 1 study focused on this score. The ACJI score includes pain, activities of daily life, cosmetic appearance, function, and imaging assessment. De Carli et al^[11]^ found that the ACJI score was 72.4 ± 1.8 and 87.9 ± 2.2 (*P* < .05) for the conservative group and surgical group, respectively, suggesting that surgical treatment was superior to conservative treatment in terms of clinical and imaging aspects. The surgical approach used in that study was the TightRope system. Salzmann et al^[22]^ applied a double TightRope device to undertake double-bundle reconstruction of the coracoclavicular ligament to treat 30 patients with acute AC dislocation, and obtained satisfactory results. Jensen et al^[23]^ treated 26 patients with acute AC dislocation using an arthroscopic double TightRope system. Of these patients, ten had type-III AC dislocation. A follow-up study of mean 17 months showed the mean visual analog score to be was 0.4, the mean Constant score to be 94, and the prevalence of complications to be 12%. With the development of minimally invasive surgery, new surgical methods may reduce the prevalence of postoperative complications.

Our meta-analysis had 4 main limitations. First, there were variations in surgical approaches and standards of functional evaluation in different studies, which could cause a bias. Second, studies written only in Chinese and English were screened and included. It is possible that some high-quality studies written in other languages were missed, which also would result in a bias in our meta-analysis. Third, studies comparing procedures involving Hook plates, ligament reconstruction, and arthroscopic surgery were lacking though new surgical methods have been developed. Such new methods may help to reduce the prevalence of postoperative complications. Finally, only 3 RCTs were included in this meta-analysis. Hence, the results of pooled analyses may include bias.

## 5 Conclusions

Traditionally, conservative procedures are the major approaches in treating AC dislocation. Such treatments cannot achieve complete anatomic reduction, but they are well accepted due to the relatively small trauma they cause, their simple procedure, and low cost. However, in recent years, with the development of minimally invasive surgery and increasing demand for reductions in treatment/recovery time, surgical treatments are being chosen (particularly by manual workers and athletes). New surgical methods may reduce the prevalence of postoperative complications. Considering the limitations of the studies reviewed, further large well-designed RCTs that include the long-term evaluation of clinically relevant outcomes in participants with various abnormal shoulder function are required to better evaluate the roles of surgical and conservative treatments in Rockwood type-III AC dislocation.

## Supplementary Material

Supplemental Digital Content
