# Supplementary material for: Comparison of surgical and conservative treatment of Rockwood type-III acromioclavicular dislocation: A meta-analysis
Source: Medicine (Baltimore). 2018 Jan 26;97(4):e9690. doi: 10.1097/MD.0000000000009690 (PMC5794375; doi:10.1097/MD.0000000000009690)
Supplement: Supplemental Digital Content [file medi-97-e9690-s001.docx]

**Search Strategies**

***A. Cochrane Library***

#1 MeSH descriptor: [Dislocations] explode all trees

#2 (Dislocat* OR Separat*):ti,ab

#3 #1 or #2

#4 MeSH descriptor: [Acromioclavicular Joint] explode all trees

#5 (Acromioclavicular OR "Acromio Clavicular"):ti,ab

#6 #4 OR #5

#7 #3 AND #6

***B. EMBASE***

1. Exp Acromioclavicular Dislocation/
2. Exp Dislocation/ OR (Dislocat* OR Separat*).ti,ab.
3. Exp Acromioclavicular Joint/ OR (Acromioclavicular OR "Acromio Clavicular").ti,ab.
4. 2 AND 3
5. 1 OR 4
6. Rockwood.ti,ab.
7. Exp Surgery/ OR (Operat* OR Surg*).ti,ab.
8. (Random* OR Factorial* OR Crossover* OR (Cross Over*) OR Cross-Over* OR Placebo* OR (Doubl* adj Blind*) OR (Singl* adj Blind*) OR Assign* OR Allocat* OR Volunteer*).mp. OR Crossover-Procedure/ OR Double-Blind Procedure/ OR Randomized Controlled Trial/ OR Single-Blind Procedure/
9. 5 AND 6 AND 7 AND 8

***C. MEDLINE***

1. Exp Dislocations/ OR (Dislocat* OR Separat*).ti,ab.
2. Exp Acromioclavicular Joint/ OR (Acromioclavicular OR "Acromio Clavicular").ti,ab.
3. Rockwood.ti,ab.
4. Exp "Surgical Procedures, Operative"/ OR (Operat* OR Surg*).ti,ab.
5. (Randomized Controlled Trial OR Controlled Clinical Trial OR Pragmatic Clinical Trial).pt. OR (Randomi?ed OR Randomly OR Placebo OR Trial OR Groups).ab. OR Drug Therapy.fs. NOT (Animals NOT (Humans AND Animals)).sh.
6. 1 AND 2 AND 3 AND 4 AND 5

***D. PubMed***

(Dislocations[Mesh] OR Dislocat*[tiab] OR Separat*[tiab]) AND ("Acromioclavicular Joint"[Mesh] OR Acromioclavicular[tiab] OR "Acromio Clavicular"[tiab]) AND Rockwood[tiab] AND ("Surgical Procedures, Operative"[Mesh] OR Surgery[Subheading] OR Operati*[tiab] OR Operate*[tiab] OR Surg*[tiab]) AND (Randomized Controlled Trial[pt] OR Controlled Clinical Trial[pt] OR Pragmatic Clinical Trial[pt] OR Randomized[tiab] OR Randomised[tiab] OR Placebo[tiab] OR Randomly[tiab] OR Trial[tiab] OR Groups[tiab]) NOT MEDLINE[sb]
